# Long-term persistence of severe acute respiratory syndrome coronavirus 2 (SARS-CoV-2) spike protein-specific and neutralizing antibodies in recovered COVID-19 patients

**DOI:** 10.1371/journal.pone.0267102

**Published:** 2022-04-21

**Authors:** Jira Chansaenroj, Ritthideach Yorsaeng, Jiratchaya Puenpa, Nasamon Wanlapakorn, Chintana Chirathaworn, Natthinee Sudhinaraset, Manit Sripramote, Piti Chalongviriyalert, Supunee Jirajariyavej, Phatharaporn Kiatpanabhikul, Jatuporn Saiyarin, Chulikorn Soudon, Orawan Thienfaidee, Thitisan Palakawong Na Ayuthaya, Chantapat Brukesawan, Duangnapa Intharasongkroh, Dootchai Chaiwanichsiri, Mila Issarasongkhram, Rungrueng Kitphati, Anek Mungaomklang, Arunee Thitithanyanont, Pijaya Nagavajara, Yong Poovorawan

**Affiliations:** 1 Faculty of Medicine, Department of Pediatrics, Center of Excellence in Clinical Virology, Chulalongkorn University, Bangkok, Thailand; 2 Medical Service Department, Bangkok Metropolitan Administration, Bangkok, Thailand; 3 Taksin Hospital, Medical Service Department, Bangkok Metropolitan Administration, Bangkok, Thailand; 4 Medical Service Department, Charoenkrung Pracharak Hospital, Bangkok Metropolitan Administration, Bangkok, Thailand; 5 Medical Service Department, Klang General Hospital, Bangkok Metropolitan Administration, Bangkok, Thailand; 6 Medical Service Department, Sirindhorn Hospital, Bangkok Metropolitan Administration, Bangkok, Thailand; 7 Medical Service Department, Ratchaphiphat Hospital, Bangkok Metropolitan Administration, Bangkok, Thailand; 8 Health Department, Public Health Center 28, Bangkok Metropolitan Administration, Bangkok, Thailand; 9 Health Department, Public Health Center 26, Bangkok Metropolitan Administration, Bangkok, Thailand; 10 National Blood Center, Thai Red Cross Society, Bangkok, Thailand; 11 Department of Disease Control, Institute for Urban Disease Control and Prevention, Ministry of Public Health, Bangkok, Thailand; 12 Faculty of Science, Department of Microbiology, Mahidol University, Bangkok, Thailand; 13 Office of the Permanent Secretary for the Bangkok Metropolitan Administration, Bangkok, Thailand; Stanford University School of Medicine, UNITED STATES

## Abstract

Understanding antibody responses after natural severe acute respiratory syndrome coronavirus 2 (SARS-CoV-2) infection can guide the coronavirus disease 2019 (COVID-19) vaccine schedule, especially in resource-limited settings. This study aimed to assess the dynamics of SARS-CoV-2 antibodies, including anti-spike protein 1 (S1) immunoglobulin (Ig)G, anti-receptor-binding domain (RBD) total Ig, anti-S1 IgA, and neutralizing antibody against wild-type SARS-CoV-2 over time in a cohort of patients who were previously infected with the wild-type SARS-CoV-2. Between March and May 2020, 531 individuals with virologically confirmed cases of wild-type SARS-CoV-2 infection were enrolled in our immunological study. Blood samples were collected at 3-, 6-, 9-, and 12-months post symptom onset or detection of SARS-CoV-2 by RT-PCR (in asymptomatic individuals). The neutralizing titers against SARS-CoV-2 were detected in 95.2%, 86.7%, 85.0%, and 85.4% of recovered COVID-19 patients at 3, 6, 9, and 12 months after symptom onset, respectively. The seropositivity rate of anti-S1 IgG, anti-RBD total Ig, anti-S1 IgA, and neutralizing titers remained at 68.6%, 89.6%, 77.1%, and 85.4%, respectively, at 12 months after symptom onset. We observed a high level of correlation between neutralizing and SARS-CoV-2 spike protein-specific antibody titers. The half-life of neutralizing titers was estimated at 100.7 days (95% confidence interval = 44.5–327.4 days, *R*^2^ = 0.106). These results support that the decline in serum antibody levels over time in both participants with severe disease and mild disease were depended on the symptom severity, and the individuals with high IgG antibody titers experienced a significantly longer persistence of SARS-CoV-2-specific antibody responses than those with lower titers.

## Introduction

The coronavirus disease 2019 (COVID-19) pandemic caused by the severe acute respiratory syndrome coronavirus 2 (SARS-CoV-2) has posed a significant threat to global public health [1,2]. The fact that highly potent SARS-CoV-2 neutralizing antibodies have been isolated from COVID-19 patients suggests that virus-specific antibodies play an important role in the protective immune response against SARS-CoV-2 infection [3]. Patients with previous episodes of COVID-19 may harbor immunoglobulins that could protect them from future infections, giving rise to the possibility of using convalescent plasma to treat COVID-19 [4,5].

Several different serological assays have been developed to estimate the longevity of antibody production and immunity against SARS-CoV-2, including lateral flow immunoassays, enzyme-linked immunosorbent assays (ELISAs), fluorescence immunoassays (FIAs), and chemiluminescence assays (CLIAs) [6]. Moreover, neutralization assays (NTs) are used to indicate whether antibodies detected after infection are indeed capable of neutralizing the virus. These assays are used for epidemiological purposes and the prediction of immunity, and usually detect anti-spike (anti-S) protein, anti-spike receptor-binding domain (anti-RBD), or the anti-nucleoprotein (anti-N) antibody response. The antibody detection rates are different, depending on other factors, such as the timing of seroconversion. The Okba N. *et al*. study demonstrated that most SARS-CoV-2 infected patients were seroconverted by two weeks after the onset of infection [7]. In addition, it was shown that IgA antibodies exhibited higher sensitivity and lower specificity than IgG, while the IgG response was longer-lived [8]. Seroconversion is typically detected between 5 and 14 days after symptom onset and persists for several months, with a median time of 5–12 days for anti-S IgM antibodies and 14 days for anti-S IgG and IgA antibodies. At the same time, the kinetics of the anti-N antibody response are like those of anti-S antibodies but may appear earlier [9–11]. Moreover, the level of neutralizing antibody in patients with severe disease developed a faster and higher-level response. It might be due to higher viral loads during SARS-CoV-2 infection overwhelming virus-induced damage in the lungs, which exacerbates proinflammatory cytokine response [12,13]. Lippi *et al*. showed that the rate of seroconversion IgG was low in patients with symptom onset less than five days while the seroconversion ranged between 15.4% and 53.8% with symptoms onset between 5 and 10 days, respectively [14]. The rate of seroconversion reached 100% for all except IgM antibodies (60%) when symptom onset occurred between 11- and 21-days post-infection. However, it is unclear whether long-term antibody persistence was associated with protective immunity.

From an immunological perspective, the durability of the antibody response is limited. Our study monitored antibody levels, including anti-S1 IgG, anti-RBD total Ig, anti-S1 IgA antibody, and neutralizing titers against wild-type SARS-CoV-2, in a longitudinal cohort of recovered COVID-19 patients for one year after symptom onset. We also evaluated the difference in serum SARS-CoV-2 antibody levels between COVID-19 patients with and without symptoms of pneumonia, based on the same classification from the previously published study [15]. An accurate quantitative assessment of the anti-SARS-CoV-2 antibody response will be essential for designing public health interventions and preventative measures, including the optimization of the COVID-19 vaccine schedule.

## Materials and methods

### Ethics statement

The study protocol was approved by the Research Ethics Committee of the Faculty of Medicine, Chulalongkorn University (Institutional Review Board [IRB] no. 572/63). Written informed consent was obtained from all participants prior to enrollment. This study was conducted from March 2020 to June 2021. We enrolled 531 individuals with virologically confirmed cases of SARS-CoV-2 infection by real-time reverse-transcription polymerase chain reaction (real-time RT-PCR) using nasal swab specimens collected at the National blood center, Thai Red Cross, Thailand (recruited from first-time plasma donors, *n* = 152), hospitals (*n* = 154), and public health centers under the Bangkok Metropolitan Administration (*n* = 225), between March and May 2020. Participants were categorized in terms of their symptom severity into those with and those without pneumonia symptoms using the definition used by the COVID-19 clinical management living guidance by World Health Organization [16]. The presence or absence of pneumonia was determined retrospectively from history taken at enrollment or patients’ medical records.

### Participants and sample collection

**Fig 1:**
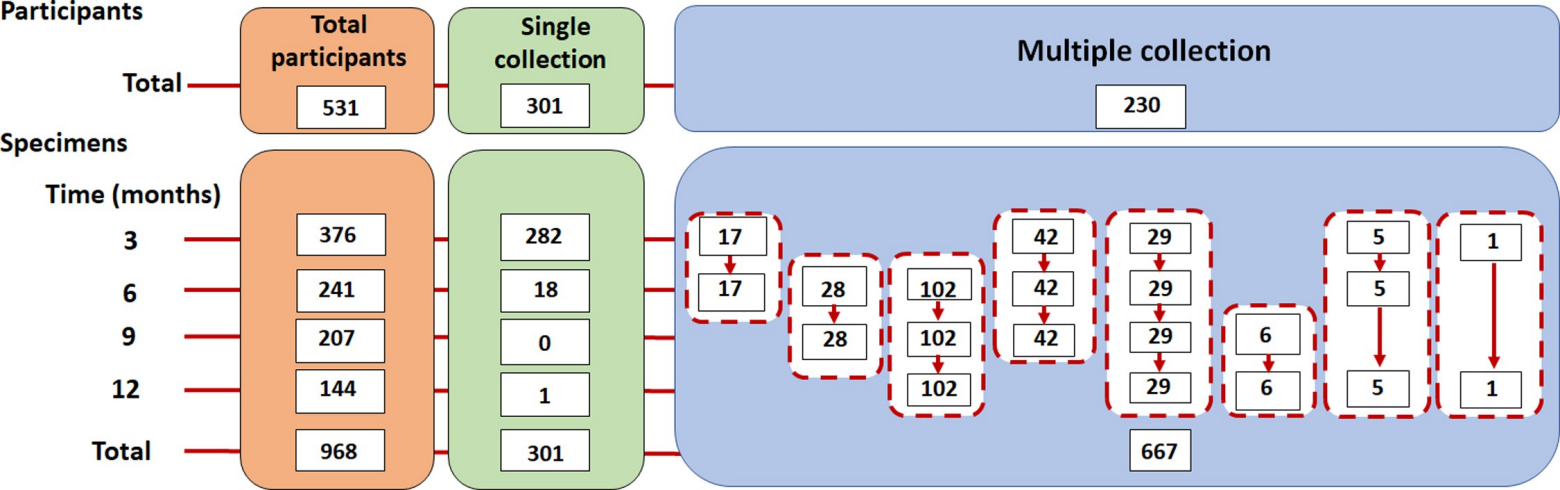
Flow diagram of participant recruitment and specimen collection in this study. A total of 531 participants were enrolled.

To investigate changes in serum SARS-CoV-2 antibody levels over time, serial blood samples from participants were collected at 3-, 6-, 9-, and 12-months post symptom onset or diagnosis. Blood was transported to the Center of Excellence in Clinical Virology Laboratory, Faculty of Medicine, Chulalongkorn University at 2–8°C within 24 hours after collection. Serum was separated from blood and kept frozen at –20°C until testing. A total of 968 specimens obtained from 531 COVID-19 patients were collected. This cohort enrolled patients diagnosed with COVID-19 infection between March and May 2020. A flow diagram of participant recruitment is shown in Fig 1. The onset date was determined as the day when the participants started experiencing COVID-19 symptoms or SARS-CoV-2 infection was confirmed by real-time RT-PCR. All patient serum samples were accompanied by information on their age, sex, symptom category (with or without pneumonia), and the symptom onset and specimen collection dates, to monitor the development of the immune response.

### Virus neutralizing assay (NT_50_)

The live virus microneutralization assay was performed as previously described [13]. Briefly, the SARS-CoV-2 virus (SARS-CoV-2/01/human/Jan2020/Thailand, Accession ID EPI_ISL_403962) was used for the *in vitro* experiments. Sera were heat-inactivated at 56°C for 30 minutes, then two-fold serially diluted starting from 1:10. Equal volumes of SARS-CoV-2 were spiked into the serial dilutions at an infectious dose of 100 TCID_50_ (50% tissue culture infectious dose), incubated for 1 hour at 37°C and transferred to the 96-well Vero E6 cells culture plates for 2 days at 37°C and 5% CO_2_. After washing, SARS-CoV/SARS-CoV-2 nucleocapsid mAb (Sino Biological, Wayne, PA) was added to each well and incubated for 2 hours at 37°C. Then horseradish peroxidase (HRP)-conjugated goat anti-rabbit polyclonal antibody (Dako, Agilent Technologies, Glostrup, Denmark) was added and incubated at 37°C for 1 hour. Next, 3,3’,5,5’-Tetramethylbenzidine (TMB) substrate was added (KPL, Seracare, Milford, MA) for 10 minutes. The reaction was stopped with 1 N HCl. Absorbance was measured at 450 and 620 nm (reference wavelength) with an ELISA plate reader (Tecan, Mannedorf, Switzerland).

The average absorbance values at 450 and 620 nm were determined for the virus and cell control wells. The neutralizing titer was expressed as the reciprocal of the highest dilution of serum that has absorbance differences between 450 and 620 nm (A_450_–A_620_) over the cut-off [17].

Sera were considered positive if the NAb titer was ≥ 20. Sera tested negative (less than 1:20 dilution) were assigned as a titer of 10.

### SARS-CoV-2 spike protein-based IgG and IgA enzyme-linked immunosorbent assays (ELISAs)

Anti-SARS-CoV-2 IgG and IgA ELISA kits (EUROIMMUN, Lubeck, Germany) were used to provide semi-quantitative *in vitro* determination of human IgG and IgA targeting the S1 domain of the SARS-CoV-2 spike protein. OD at 450 nm was measured. The results can be evaluated semi-quantitatively by calculating the ratio of the extinction of the control or patient sample over the extinction of the calibrator. Samples with a cutoff ratio were classified into the three categories: positive (ratio > 1.1), borderline (0.8 ≤ ratio ≤ 1.1), or negative (ratio < 0.8). All ELISAs were tested automatically using the EUROIMMUN Analyzer I-2P machine.

### Electrochemiluminescence immunoassay (ECLIA)

The Elecsys Anti-SARS-CoV-2 S (Roche diagnostics GmbH, Mannheim, Germany) is an electrochemiluminescence immunoassay intended for the qualitative and semi-quantitative detection of antibodies against SARS-CoV-2. This assay uses a recombinant protein representing the receptor-binding domain (RBD) of the spike antigen in a double-antigen sandwich assay format. The antigens within the reagent capture predominantly anti-SARS-CoV-2 IgG, but also anti-SARS-CoV-2 IgA and IgM. The test is intended for use as an aid for identifying individuals with an adaptive immune response to SARS-CoV-2, indicating recent or prior infection. The analyzer automatically calculates the analyzed concentration of each sample in U/ml. A result < 0.8 U/ml represents ‘negative for anti-SARS-CoV-2’ and ≥ 0.8 represents ‘positive for anti-SARS-CoV-2’.

### Statistical analysis

All Statistical analyses were performed using IBM SPSS Statistics for Windows, version 21 (IBM Corp., Armonk, NY) and GraphPad Prism version 9.0 software (GraphPad, San Diego, CA). Descriptive statistics were used to analyze participants’ characteristics. The median (interquartile range, IQR) was used for continuous variables with a skewed distribution. The geometric mean of the neutralizing titers was calculated by multiplying all the numbers in the group and take the n^th^ root for the obtained result. The difference between groups was examined by Student’s *t*-test or Mann-Whitney U test, as appropriate. For categorical variables, the Chi-squared test or Fisher’s exact test was used. The association between the seropositivity rate of SARS-CoV-2 antibodies and disease severity was analyzed using the Chi-squared test. Spearman rank-order correlation analysis was performed to evaluate the relationship between neutralizing titer and immunoassays. Non-linear regression analysis gave a measure of the regression correlation between the neutralizing titers and interval time after symptoms onset. A *p*-value < 0.05 was considered statistically significant.

## Results

### Participant characteristics

To investigate antibody responses toward SARS-CoV-2 over time, recovered COVID-19 patients were recruited into the longitudinal study. The participants had a follow-up visit every 3 months for 12 months after disease onset to perform a longitudinal analysis of IgG and IgA using various immunoassays. 968 serum specimens were obtained from 531 participants between March 2020 and June 2021, following the previous study [18]. The specimens were classified into four time-ranges after symptom onset or diagnosis: 3 months (median 56 days after positive real-time RT-PCR/symptoms, *n* = 376), 6 months (median 204 days after positive real-time RT-PCR/symptoms, *n* = 241), 9 months (median 291 days after positive real-time RT-PCR/symptoms, *n* = 207), and 12 months (median 372 days after positive real-time RT-PCR/symptoms, *n* = 144). The baseline demographics of these 531 participants are described in Table 1. The study group was 50.6% (269/531) males and 49.3% (262/531) females, with an age range of 2–82 years (median, 36 years). A significant difference was found in the comparison of disease severity and age (*p*-value < 0.01), but no significant difference was found in the comparison of disease severity and sex (*p*-value = 0.357). All analyzed participants in this study were also classified according to their symptoms: 111 with pneumonia (‘with pneumonia’ group) and 420 without pneumonia (‘without pneumonia’ group).

**Table 1:** Demographic data of participants in this study.

Participants	Characteristic	Symptoms		*p*- value
		Without pneumonia,	With pneumonia,	
		N = 420	N = 111	
Age, years	Median age (IQR)	35 (26.5–44.0)	39 (32.0–50.0)	<0.01
	Mean age (SD)	36.8 (11.9)	40.9 (13.1)	
Age, years	<20 (N, %)	11 (2.6)	1 (1.0)	
	20–39 (N, %)	253 (60.2)	56 (50.5)	
	40–59 (N, %)	132 (31.4)	42 (37.8)	
	>59 (N, %)	17 (4.0)	12 (10.8)	
	Unknown (N, %)	7 (1.7)	0 (0.0)	
Sex	Male (N, %)	209 (49.8)	60 (54.1)	0.357
	Female (N, %)	211 (50.2)	51 (45.9)	

Abbreviations: IQR, Interquartile range; SD, Standard deviation.

### Serological outcomes

**Fig 2:**
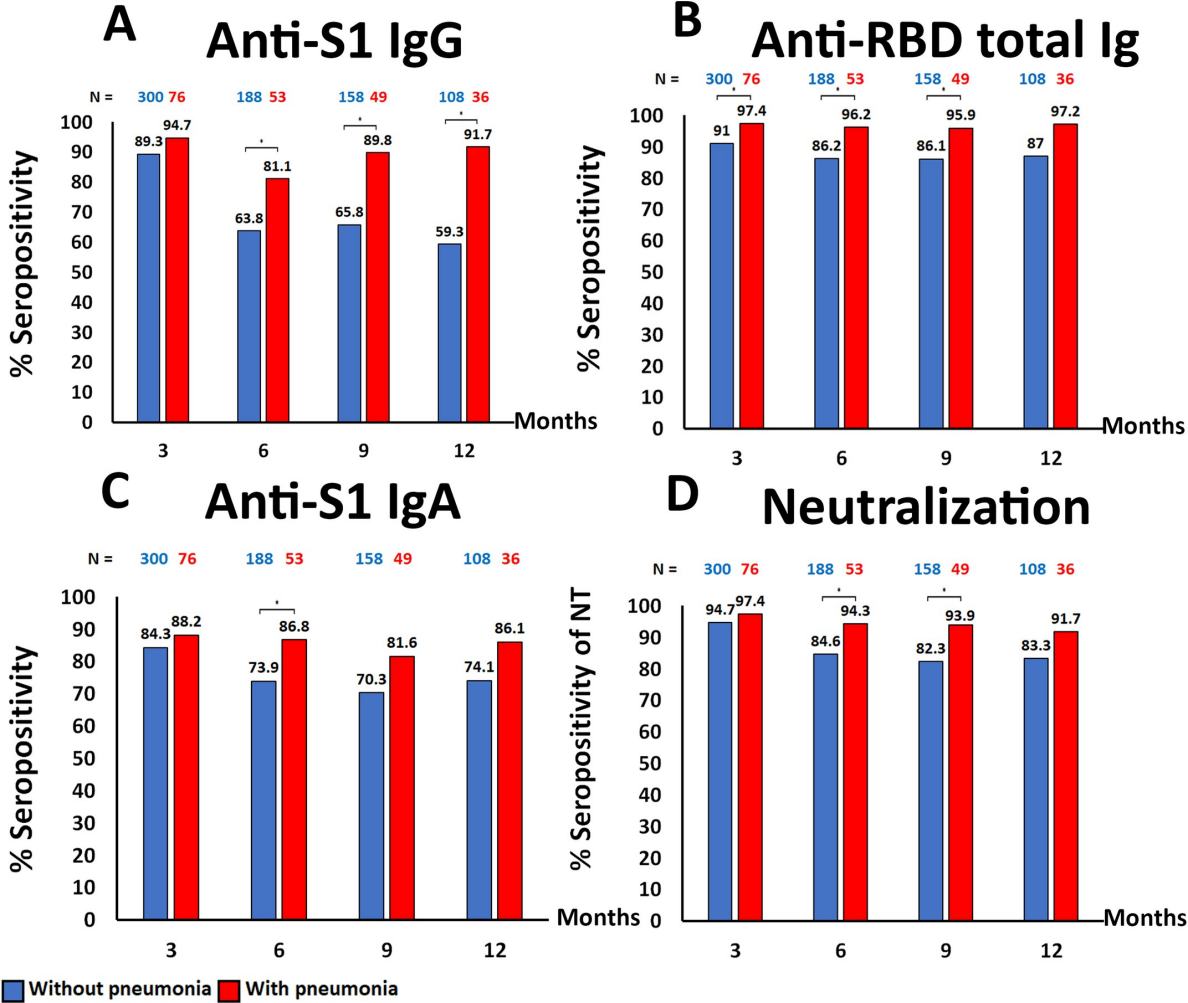
Comparison of seropositivity rate among recovered COVID-19 patients with pneumonia (red) and without pneumonia (blue) at indicated time points after post symptom onset or first SARS-CoV-2 detection by using Chi-square test, (* = *p*-value < 0.05). (A) Anti-S1 IgG, (B) Anti-RBD total Ig, (C) Anti-S1 IgA, (D) Neutralizing titer, measured by the virus-neutralizing assay (NT_50_).

The seropositivity rate of the samples collected at 3, 6, 9, and 12 months after diagnosis was analyzed (Fig 2). The anti-S1 IgG was detected in 89.3% (268/300), 63.8% (120/188), 65.8% (104/158), and 59.3% (64/108) of samples from patients without pneumonia, and 94.7% (72/76), 81.1% (43/53), 89.8% (44/49), and 91.7% (33/36) of samples from patients with pneumonia, at 3, 6, 9, and 12 months after diagnosis, respectively. The anti-RBD total Ig was detected in 91.0% (273/300), 86.2% (162/188), 86.1% (136/158), and 87.0% (91/108) of samples from patients without pneumonia, and 97.4% (74/76), 96.2% (51/53), 95.9% (47/49), and 97.2% (35/36) of samples from patients with pneumonia, at 3, 6, 9, and 12 months after diagnosis, respectively. The anti-S1 IgA was detected in 84.3% (253/300), 73.9% (139/188), 70.3% (111/158), and 74.1% (80/108) of samples from patients without pneumonia, and 88.2% (67/76), 86.8% (46/53), 81.6% (40/49), and 86.1% (31/36) of samples from patients with pneumonia, at 3, 6, 9, and 12 months after diagnosis, respectively. For all antibody titers tested in this study, the seropositivity rate declined at the 6 months after diagnosis. In samples from patients without pneumonia, the seropositivity rate was lower than those with pneumonia at all time-points. The NT seropositivity rates were 94.7% (284/300), 84.6% (159/188), 82.3% (130/158), and 83.3% (90/108) in samples from patients without pneumonia, and 97.4% (74/76), 94.3% (50/53), 93.9% (46/49), and 91.7% (33/36) in samples from patients with pneumonia, at 3, 6, 9, and 12 months after diagnosis, respectively. The geometric mean of neutralizing antibody titer at all time-points was significantly different between pneumonia and non-pneumonia/asymptomatic COVID-19 patients (330.6 vs 144.7, *p*-value < 0.01).

### Long-term antibody titers

**Fig 3:**
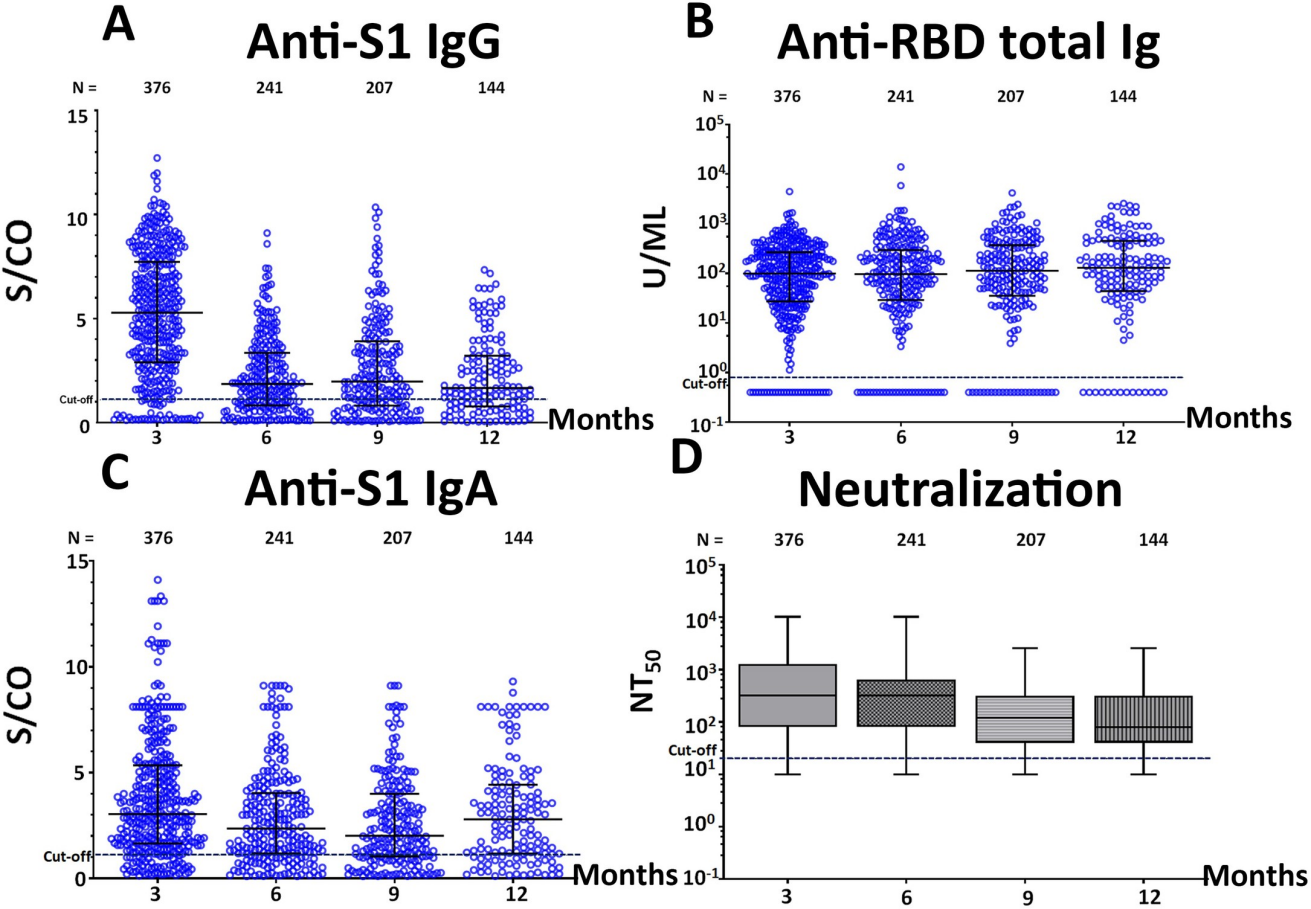
The comparison between the antibody level of all specimens in this study. (A) Anti-S1 IgG, (B) Anti-RBD total Ig, (C) Anti-S1 IgA, (D) Neutralizing titer (NT_50_).

**Fig 4:**
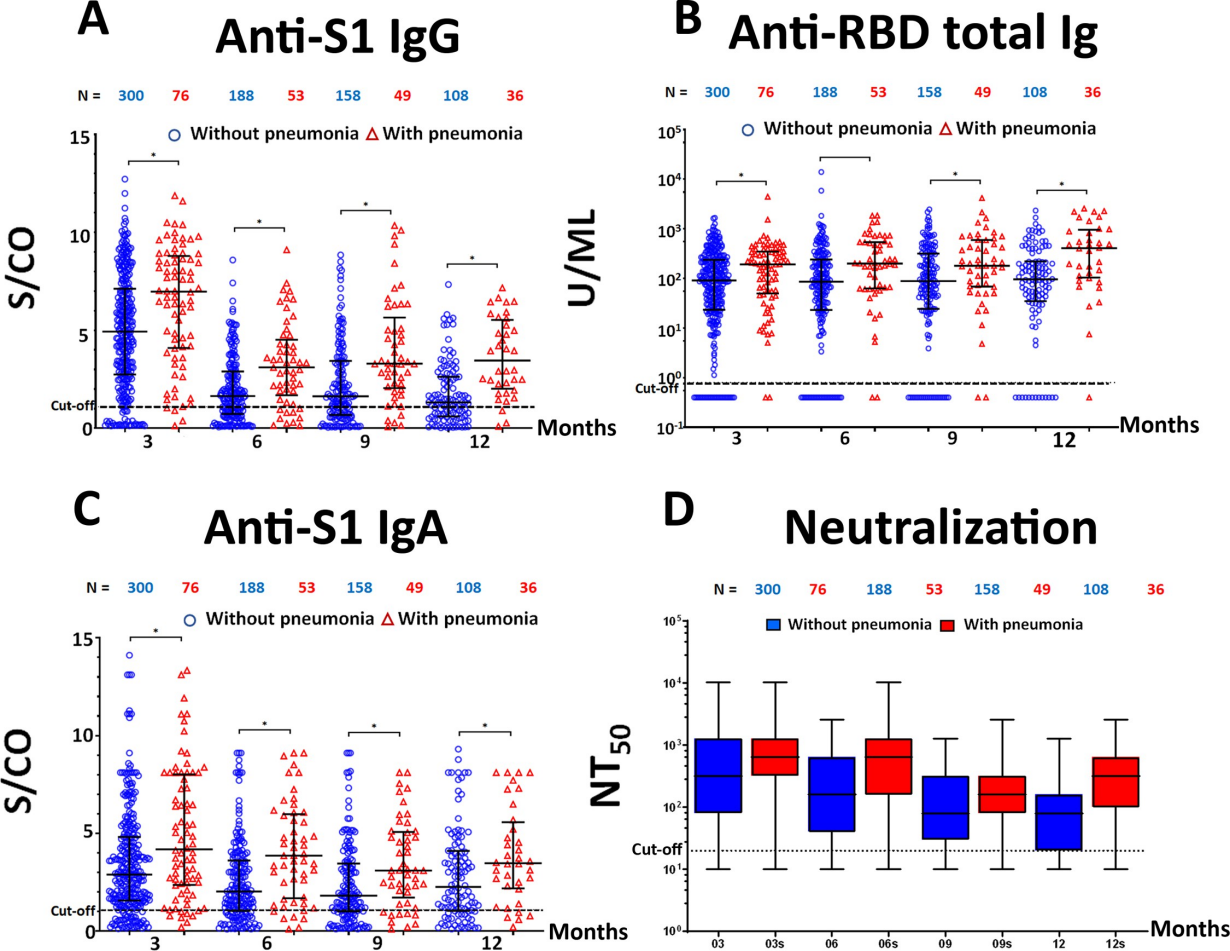
The comparison of antibody levels in the ‘with pneumonia’ (red) and ‘without pneumonia’ (blue) study groups, (* = *p*-value < 0.05). (A) Anti-S1 IgG, (B) Anti-RBD total Ig, (C) Anti-S1 IgA, (D) Neutralizing titer (NT_50_).

When classified according to the presence or absence of pneumonia, the anti-S1 IgG, anti-S1 IgA antibody titer and neutralization titers of all determinations showed a significant reduction of the antibody titers over time except for anti-RBD total Ig (Figs 3 and 4). We also determined the dynamics of specific antibody titers 12 months after symptom onset. The median and geometric mean titer (GMT) of antibody titers is shown in S1 Table. The anti-S1 IgG, anti-S1 IgA, and neutralizing antibody titers against SARS-CoV-2 peaked a few months after infection, which was followed by a contraction phase lasting several months. Stabilized antibody responses could be detected for over 12 months. Only the anti-RBD total Ig assay showed a tendency toward an increase in antibody titer for over 12 months.

The results of Spearman’s correlation analysis demonstrated a statistically significant positive relationship between neutralizing antibody titers and anti-S1 IgG, anti-RBD total Ig, and anti-S1 IgA levels; in the ‘without pneumonia’ group: *r*_*s*_ = 0.73, *p*-value < 0.001; *r*_*s*_ = 0.67, *p*-value < 0.001; *r*_*s*_ = 0.59, *p*-value < 0.001, respectively, and in the ‘with pneumonia’ group: *r*_*s*_ = 0.62, *p*-value < 0.001; *r*_*s*_ = 0.53, *p*-value < 0.001; *r*_*s*_ = 0.50, *p*-value < 0.001, respectively (S1 Fig).

**Fig 5:**
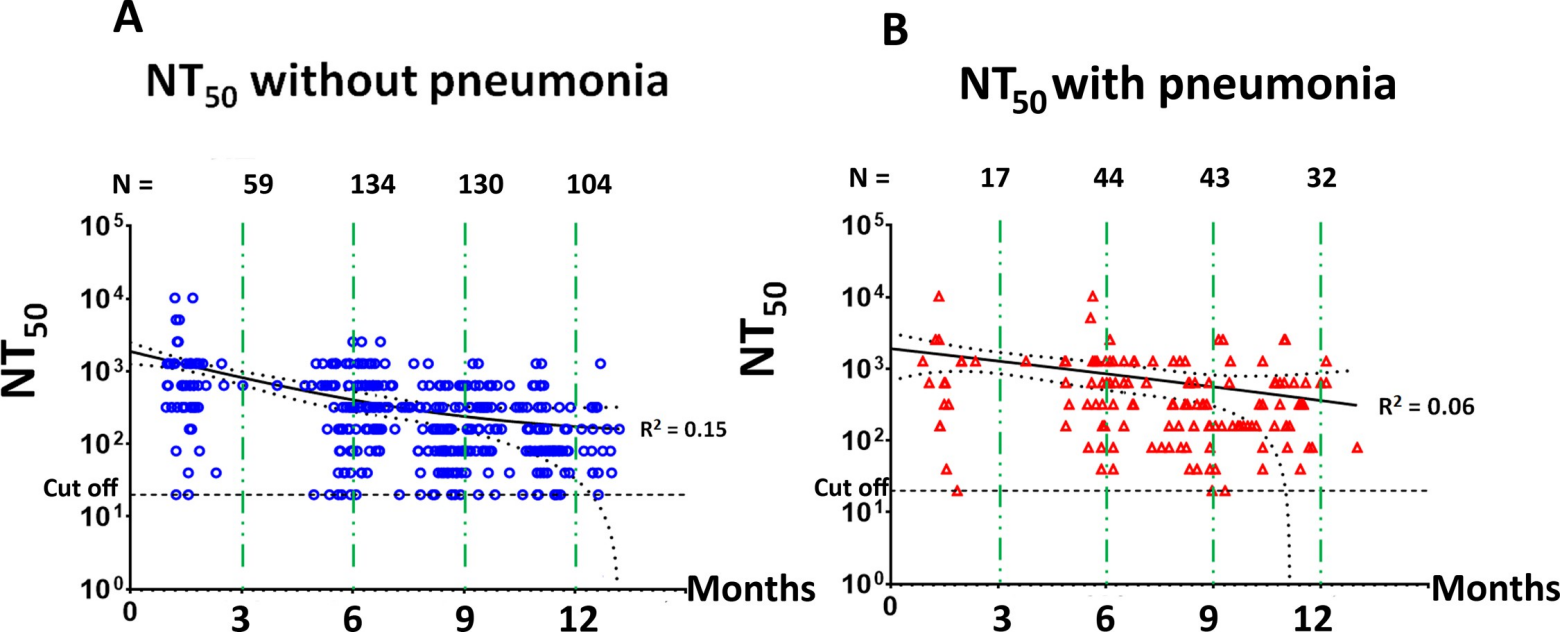
SARS-CoV-2 neutralizing titer in a longitudinal cohort of recovered COVID-19 patients who provided blood samples for at least three time-points. **(**A) in the ‘without pneumonia’ group, (B) in the ‘with pneumonia’ group.

Neutralizing antibody titers against wild-type SARS-CoV-2 in a longitudinal cohort of recovered COVID-19 patients who provided blood samples for at least three time-points were plotted over time (Fig 5). The non-linear regression, one-phase decay model predicted a neutralizing titer half-life of 74.9 days in the ‘without pneumonia’ group (95% confidence interval = 26.4–185.1 days, *R*^2^ = 0.15) and 181.3 days in ‘with pneumonia’ group (95% confidence interval = 10.44–421.1 days, *R*^2^ = 0.06).

We next evaluated whether COVID-19 disease severity or sex correlated with the magnitude of the SARS-CoV-2-specific antibody immune response. The results showed that anti-S1 IgG, anti-RBD total Ig, anti-S1 IgA, and neutralizing antibody titers appeared higher in the ‘with pneumonia’ group when compared with the ‘without pneumonia’ group. The increase in disease severity was significantly associated with a stronger immune response to SARS-CoV-2 (*p*-value < 0.01). However, no significant relationship between sex and immune response magnitude was observed. (S2 Fig)

## Discussion

Establishing an immune response is essential in the defense against SARS-CoV-2 infection. To end the COVID-19 pandemic, it is critical to know how long immunity against SARS-CoV-2 will persist after infection and whether it will be sufficient to prevent re-infection. Although several COVID-19 vaccines currently show promising efficacy in preventing SARS-CoV-2 infection and inducing anti-viral antibodies [19–22], there is still no consensus regarding vaccine schedules for individuals with a previous history of SARS-CoV-2 infection, due to limited information about immune responses after natural infection [23,24]. Therefore, longitudinal studies of natural infection provide valuable insights into the kinetics and durability of protective immune responses, with the aim of improving vaccination strategy.

Many studies have supported the notion that IgG, and IgA titers are higher in severely and critically ill COVID-19 patients, often associated with complex immune dysregulation, CD4 cytopenia, and macrophage activation [25–27]. In the present study, antibodies against SARS-CoV-2, including IgG and IgA, were comprehensively investigated in individuals with COVID-19 to delineate their relationship with disease severity. Commercial automated high-throughput SARS-CoV-2 immunoassays performed on samples from recovered COVID-19 participants have revealed that anti-N IgG titers peak in the third month post infection and gradually wane to undetectable within 6 months after symptom onset [18]. Meanwhile, high titers of anti-S1 IgG and IgA can be detected during 6 months after symptom onset, then drop slightly and remain present over 12 months after infection. The results indicate that anti-S1 IgG and IgA titers may stabilize following the infection period, while anti-N IgG levels increase immediately after SARS-CoV-2 infection but decline soon after, with a much shorter half-life. Likewise, in previous studies, COVID-19 infected individuals became seronegative for anti-N within a few months of SARS-CoV-2 infection, while anti-S1 IgG and IgA titers decayed slowly and remained detectable over 6 months post symptom onset [28–30].

Normally, higher antibody titers correlate with worse clinical readouts and older age, suggesting the potentially detrimental effects of antibodies in some patients [31]. The IgG response is typically longer lasting to help fight off infection, and high IgG titers in a patient’s blood can indicate a later infection stage. Moreover, individuals with high IgG antibody titers have been shown to experience a significantly longer duration of COVID-19 than those with low titers [32]. It suggests that a longer COVID-19 course is associated with the elevated production and persistence of certain SARS-CoV-2-specific antibody subsets.

In the present study, we found that the increase in disease severity was significantly associated with a stronger antibody-mediated immune response to SARS-CoV-2 (*p*-value < 0.01). Many previous studies have supported this finding. For instance, Tay *et al*. showed that neutrophilia and an increase in the neutrophil/lymphocyte ratio in COVID-19 patients were usually accompanied by advanced disease severity and poor clinical outcome [33]. Meanwhile, Huang *et al*. [34] found that the most severely COVID-19 patients experienced a cytokine storm (CS), characterized by the presence of higher levels of proinflammatory cytokines in the serum [35]. Therefore, the measurement of anti-S IgG levels can be a reliable and convenient tool for assessing the immunological response of COVID-19-infected individuals, to quantify the immunogenicity of vaccines and therapeutic efforts [36,37].

The anti-RBD total Ig assay, measuring IgG, IgM, and IgA isotypes, showed sustained total Ig levels even if the titers of individual isotypes declined over the same period. This result is in concordance with reports which describe rising total antibody levels over time, using pan-immunoglobulin assays; titers rose for two months and then reached a plateau for at least another two months, in contrast to the declining isotype-specific SARS-CoV-2 antibodies, is maintained at least for three months [38–40]. A previous study showed that RBD-specific memory B cell numbers were unchanged while anti-N IgG titers sharply decayed, with only 20% of individuals remaining seropositive after one year post SARS-CoV-2 infection. This difference could be explained by an increase in avidity that compensates for antibody loss or changes in recognized epitopes over time. Memory B cells display clonal turnover 6.2 months after infection, following which the antibodies they express acquire more somatic hypermutations, increased potency, and resistance to RBD mutation, indicative of continued evolution of the humoral response [38,41]. However, how long these antibodies persist in the body or whether patients who have developed an antibody response to SARS-CoV-2 are protected from re-infection, remains unknown. The emerging data suggest that acquired immunity following primary SARS-CoV-2 infection offers protection from re-exposure [10,42]. The persistence of antibodies is unlikely to be the sole determinant of long-lasting immunity, with the anamnestic recall of stably maintained antibody populations likely reducing infection or disease severity. The magnitude, quality, and protective potential of cellular responses against SARS-CoV-2, therefore, require further definition [43].

The role of serum IgA is relatively unexplored in contrast with mucosal IgA. Previous studies have shown that IgA exerts either pro- or anti-inflammatory effects on innate immune cells by downregulating proinflammatory cytokine or upregulating anti-inflammatory cytokine expression by peripheral blood mononuclear cells (PBMCs) [44,45]. The monomeric binding of serum IgA to the Fc alpha receptor (FcαRI) has been suggested to have an inhibitory function via the transmission of inhibitory signals in a variety of myeloid cells [46]. IgA likely acts as a driver of autoimmune disease and as a regulator of immune hyperactivation [47]. Due to a regulator of immune hyperactivation, this may be influenced by more disease severity in the patients. Therefore, IgA is a good surrogate marker to predict the clinical course of COVID-19. In addition, a previous study reported that early baseline antibody levels were key drivers of the subsequent antibody production and the long-lasting protection against SARS-CoV-2 [48–50]. In accordance with the previous studies [48,49], we found that the level of anti-S1 IgA in COVID-19 patients was relatively high and was maintained over 9 months after infection (in over 70% and 80% of patients without pneumonia and with pneumonia, respectively).

The modeled half-life of anti-N IgG is approximately 60 days (which is shorter than that of anti-S IgG, anti-RBD total Ig, and anti-S IgA) was predicted to remain detectable in over 50% of study participants until 12 months post SARS-CoV-2 infection [28]. The neutralizing antibody titer half-life in a longitudinal cohort of recovered COVID-19 patients, who provided blood samples for at least three time-points, was estimated at 100.7 days, like a previous report showing that neutralizing responses decay slowly, persisting for 90–150 days after infection [51].

Importantly, the antibody titer examines the infection severity and the chance of a successful recovery and determines whether herd immunity has been reached among the population. Although our study revealed the association between antibody levels and disease severity, the amount of viral load in the study subjects was not measured. Therefore, high antibody titers may also facilitate viral clearance. Longitudinal studies will be required to determine the longevity and the dynamics of the antibody response, to identify risks and develop interventions aimed at minimizing disease transmission.

Due to the limitations of this study, such as the low number of clinical specimens covering all four time-points (i.e., data from > 2 time-points were collected for only 177 participants), it is difficult to determine a clear association between the antibody response and disease severity. However, our study offers valuable insights into the long-term humoral immune response against SARS-CoV-2 infection. These data may therefore have implications for COVID-19 vaccine development and implementation, as well as other public health responses to the COVID-19 pandemic. However, longer follow-up studies are needed to determine the durability of these responses and their correlations with clinical protection.

In summary, we showed that antibody titers are associated with disease severity and interval between symptom onset and blood sampling. However, the persistence of anti-S1 IgG and IgA in recovered COVID-19 patients was observed to last longer than 12 months after symptom onset, while the anti-N IgG response disappeared almost entirely 6 months after symptom onset. These results may apply to the strategic planning of serological diagnosis, vaccine development, immunization, and decision-making in terms of social-economic mitigation.

## Supporting information

S1 Fig The correlation between neutralizing antibody titers and anti-S1 IgG, anti-RBD total Ig, and anti-S1 IgA levels in the ‘with pneumonia’ (red) and ‘without pneumonia’ (blue) study groups. (TIF)

S2 Fig The comparison of antibody levels in the ‘with pneumonia’ (red) and ‘without pneumonia’ (blue) study groups classified by gender, (* = *p*-value < 0.05). (A) Anti-S1 IgG, (B) Anti-RBD total Ig, (C) Anti-S1 IgA, (D) Neutralizing titers (NT_50_).
(TIF)

S1 Table The percentage of seropositivity, median and GMT of Anti-S1 IgG, Anti-RBD total Ig, Anti-S1 IgA, Neutralizing titers. (XLSX)
