# Supplementary figures and images for: Long-term persistence of severe acute respiratory syndrome coronavirus 2 (SARS-CoV-2) spike protein-specific and neutralizing antibodies in recovered COVID-19 patients
Source: PLoS One. 2022 Apr 21;17(4):e0267102. doi: 10.1371/journal.pone.0267102 (PMC9022880; doi:10.1371/journal.pone.0267102)

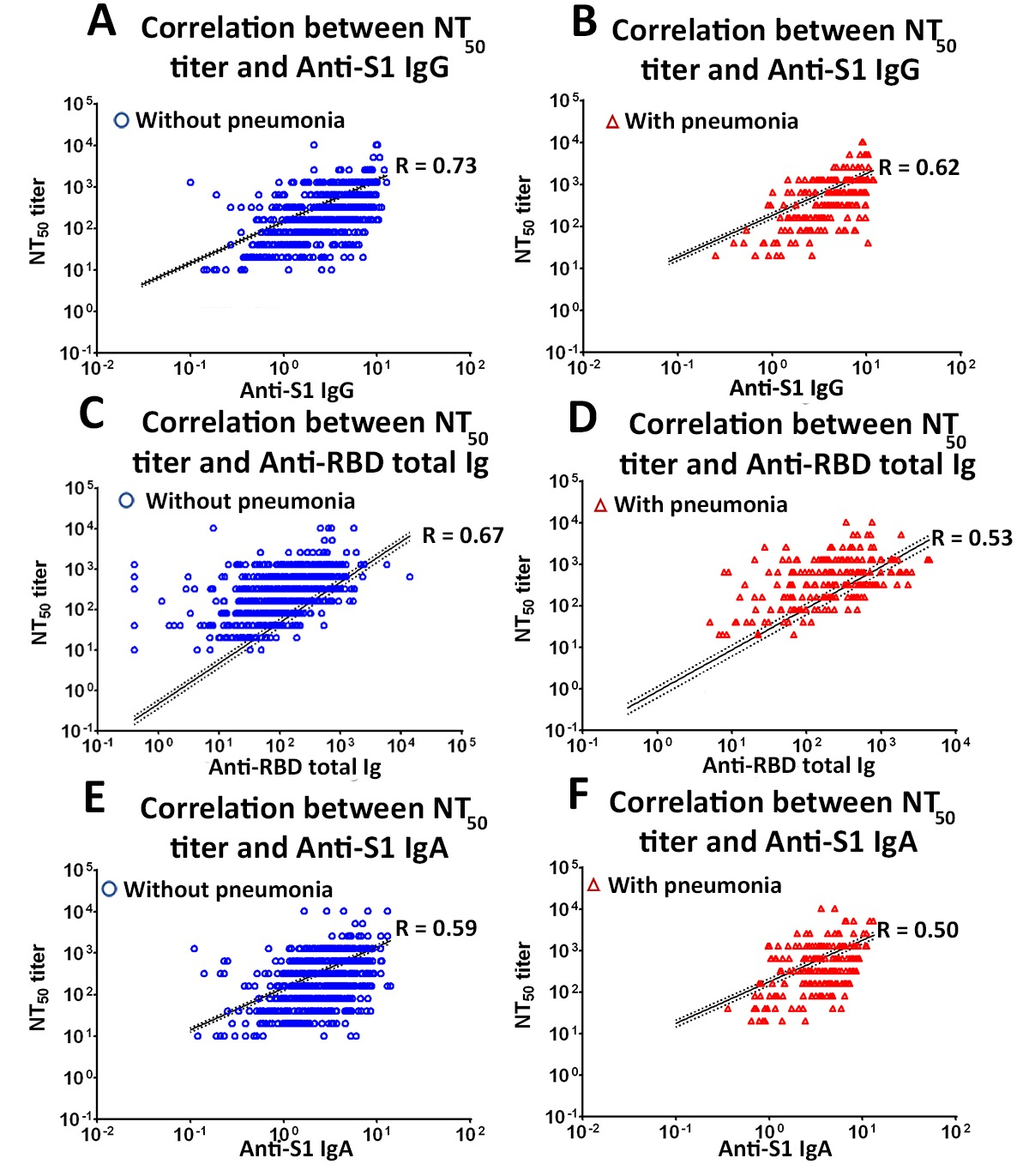

Supplement: S1 Fig — (TIF) [file pone.0267102.s001.tif]

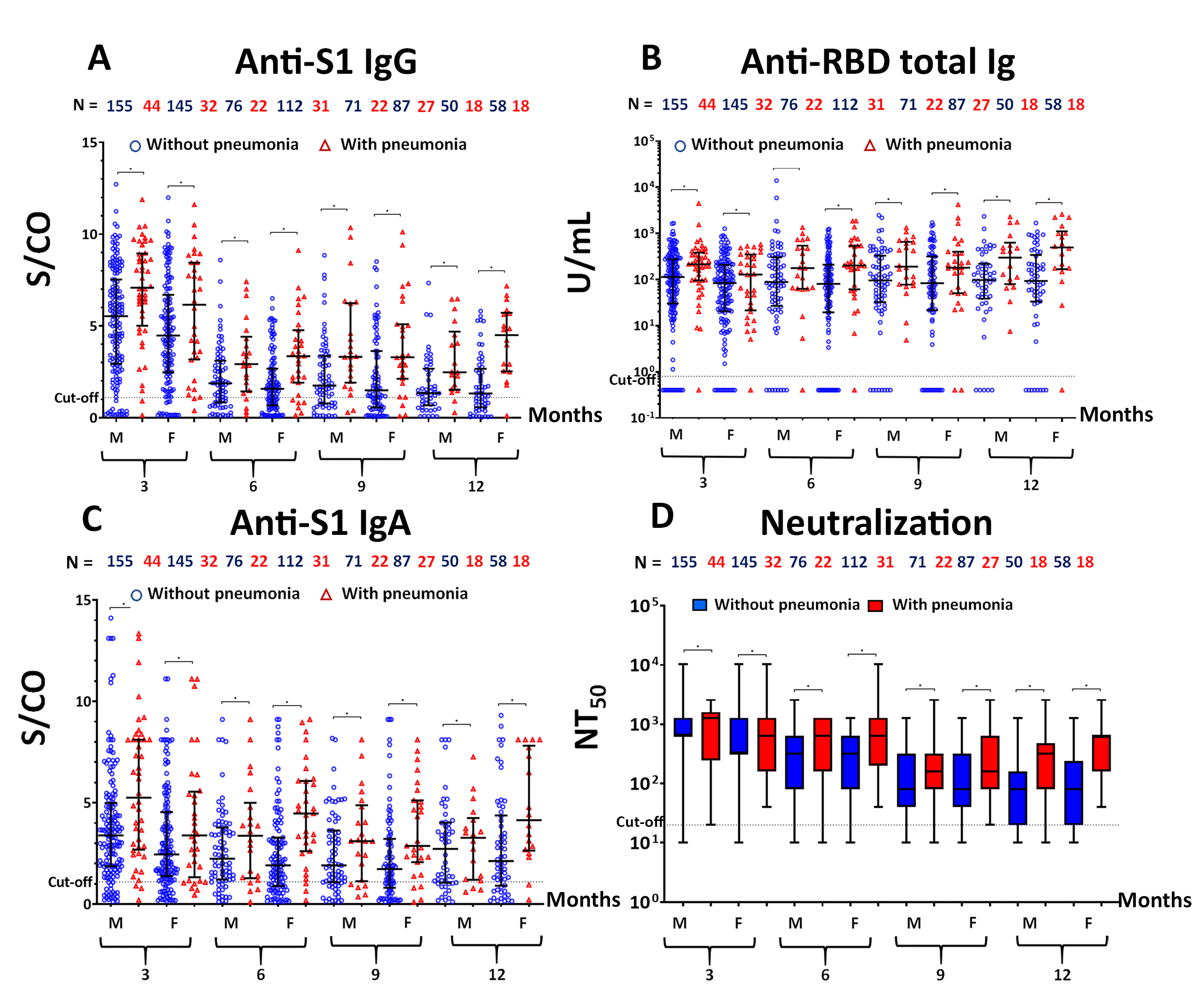

Supplement: S2 Fig — (A) Anti-S1 IgG, (B) Anti-RBD total Ig, (C) Anti-S1 IgA, (D) Neutralizing titers (NT50). (TIF) [file pone.0267102.s002.tif]
